# The Suitability of Questionnaires for Exploring Relations of Dietary Behavior and Tooth Wear

**DOI:** 10.3390/nu14061165

**Published:** 2022-03-10

**Authors:** Maximiliane Amelie Schlenz, Moritz Benedikt Schlenz, Bernd Wöstmann, Alexandra Jungert, Anna Sophia Glatt, Carolina Ganss

**Affiliations:** 1 Department of Prosthodontics, Dental Clinic, Justus Liebig University, Schlangenzahl 14, 35392 Giessen, Germany; maximiliane.a.schlenz@dentist.med.uni-giessen.de (M.A.S.); moritz.schlenz@dentist.med.uni-giessen.de (M.B.S.); bernd.woestmann@dentist.med.uni-giessen.de (B.W.); 2 Biometry and Population Genetics, Institute of Agronomy and Plant Breeding II, Interdisciplinary Research Center for Biosystems, Land Use and Dietary (IFZ), Justus Liebig University, Heinrich-Buff-Ring 26-32, 35392 Giessen, Germany; alexandra.jungert@ernaehrung.uni-giessen.de; 3 Department of Conservative and Preventive Dentistry, Dental Clinic, Justus Liebig University, Schlangenzahl 14, 35392 Giessen, Germany; anna.s.glatt@dentist.med.uni-giessen.de

**Keywords:** diet, oral health, questionnaires, erosion, young adults, tooth wear, dentistry

## Abstract

Tooth wear is a relevant oral health problem, especially at a young age. Although ongoing acid exposures may contribute to tooth wear, the role of acidic dietary components in this context remains unclear. To date, in tooth wear studies, dietary behavior has been assessed using traditional questionnaires, but the suitability of this approach has not been investigated so far. In our longitudinal study, we followed 91 participants (21.0 ± 2.2 years) over a period of 1 year (373 ± 19 days) and monitored tooth wear with an intraoral scanner. At baseline (T0) and at the end (T1), we assessed dietary behavior with questionnaires asking about the consumption frequencies of acidic dietary components and the acid taste preferences. Complete data were available from 80 subjects. The consumption frequencies of T0 and T1 correlated weakly to moderately. Taste preferences seem to be a more consistent measure, but there was predominantly no significant correlation with the corresponding consumption frequencies. None of the dietary parameters showed a significant relation with tooth wear. The suitability of dietary questionnaires to assess tooth-relevant dietary behavior seems to be limited.

## 1. Introduction

Tooth wear is a type of dental hard tissue loss that occurs due to the effects of physical and chemical factors. Physical factors include forces due to tooth-to-tooth contact (attrition) that can appear as parafunctions, such as tooth grinding. Furthermore, physical factors can also be so-called foreign objects (abrasion), for example, oral hygiene may play a role. Chemical factors include acid exposure of any kind that does not originate from bacterial metabolism (erosion) [1]. These factors affect the dental hard tissue throughout the entire functional period of the dentition and cause irreversible and, therefore, cumulative dental hard tissue loss.

Enamel is a very densely mineralized avital tissue with considerable hardness and wear resistance [2]. However, acid impacts are able to demineralize enamel relatively easily, which can significantly reduce its microhardness. As a result, subsequent physical impacts may increase dental hard tissue loss [3]. All these factors interact with the multiple functions of the oral cavity. When acid impacts are prominent, it is referred to as erosive tooth wear [1].

That a variety of food and drinks are capable of demineralizing enamel is well known from laboratory experiments [4]. These include, for example, fruit juices, soft drinks, wines, fruit, vinegar, and pickles. It is, therefore, plausible to assume that an acidic diet can contribute to tooth wear. However, epidemiological studies show only weak associations, if any, between acid impacts from the diet and the prevalence and severity of tooth wear. For example, a systematic review that included more than fifty cross-sectional studies had very heterogeneous and contradictory results, with the majority of studies showing no association between dietary acid impacts and tooth wear [5].

While cross-sectional studies can, at best, show associations, longitudinal surveys are more capable of identifying causal or risk factors, but well-conducted studies are sparse. A Dutch study [6] included 656 children aged 10–12 years, of whom 572 were followed up 3 years later. The only risk factors identified were frequent consumption of alcoholic mixed drinks and acidic vegetables. However, the strength of the association was weak (alcoholic mixed drinks OR = 1.82; acidic vegetables OR = 1.16). A recent Brazilian study [7] included 801 12-year-olds; 680 could be followed up after 2 and a half years. Dietary behavior was not a risk factor in this study.

Generally, in such studies, the dietary data are collected only once, in longitudinal studies at the beginning of the study period. Furthermore, the consumption frequencies of individual items or individual classes (e.g., soft drinks or fruit juices) are usually considered, which means that the total amount of daily acid impacts, regardless of their source, is not taken into account. To the best of the authors’ knowledge, only two studies also included a measure for the total amount of acidic drinks consumed [8,9].

Even if these methodological aspects can, at least, partially explain the heterogeneous study results, the question remains: why it is so difficult to establish associations between dietary behavior and tooth wear, even though the potential demineralizing properties of acidic food have been clearly demonstrated? It is conceivable that questionnaires do not adequately reflect actual eating behavior, that a cross-sectional observation of diet is not very representative of dietary behavior over a longer period of time, or that dietary behavior is so variable that individual surveys generally have little significance when it comes to processes that only manifest themselves in longer contexts.

For this reason, we have modified the above-mentioned mostly used procedure for collecting dietary data. When such data are collected at the beginning of a longitudinal study, it is assumed that these data will reflect dietary behavior over the course of the observation period. However, since this assumption cannot be easily substantiated, we conducted a second survey at the end of the observation period. Furthermore, we did not only collect data on the consumption frequencies of acidic food and drinks but also the taste preferences of our study participants.

The main aim of our study was to investigate to what extent the dietary habits reported at baseline are related to those reported at the end of the observation period and whether the reported taste preferences reflect the reported consumption frequencies. A further but subordinate aim was to explore how these results relate to tissue loss values measured with an intraoral scanner in the corresponding observation period.

## 2. Participants, Materials and Methods

### 2.1. Study Group

The study was conducted in accordance with the principles of the Declaration of Helsinki [2] and approved by the Ethics Committee of the medical faculty of the Justus Liebig University Giessen (ref. no. 148/18). The procedure and methodology of the study have been described earlier [10] as part of the project “Intraoral scanner-based monitoring of tooth wear in young adults”. The data for this publication were based on the data set from [10].

Between the end of 2018 and the beginning of 2020, a total of 91 participants (mean age at the start of the study: 21.0 ± 2.2 years) were included. Inclusion criteria were defined as age between 18 and 25 years, a lower first molar without caries and clinically visible plaque, as well as without any restoration or one whose extent did not exceed one-third of the occlusal width. The same criteria were defined for the occluding antagonist. Exclusion criteria were determined as severe disease [11], ongoing orthodontic treatment, and maxillary crowns or bridges.

### 2.2. Questionnaire

The questionnaire regarding dietary behavior was designed by an experienced nutritionist (A.J.) and provided in paper form. In advance of this study, cognitive pre-tests using the technique of thinking aloud were applied, and expert interviews were conducted. The survey contained questions about the frequency of consumption of specific drinks and food items in order to calculate the acid impacts per day. To this end, for each frequency category, the mean value of the given range was determined and subsequently converted in frequencies per day. As an example: for the consumption frequency “1–4× per week”, the mean would be 2.5× per week, converted to the day this would result in 0.36. The values of all items (or those related to food or drinks separately) were summed and constituted the total number of daily acid impacts (or acid impacts from drinks and food, respectively).

Furthermore, a five-point Likert scale was used to ask about the taste preference for drinks and food. The abstention of answers was allowed, and questionnaires were evaluated anonymously. The items of the questionnaire that were analyzed here can be found in the Appendix A. Participants completed a dietary questionnaire twice: at baseline (T0) and at follow-up after one year (T1; mean observation time 373 ± 19 d).

Please also see further information in the Appendix A.

#### Re-Test of Questionnaire

To investigate the reliability of the questionnaire, a re-test was conducted independently of the main study procedure with a group of 28 participants (12 female, 16 male; age 25.8 ± 4.6 years) not involved in the clinical study. Participants were asked to fill out the questionnaire twice at a 1-week interval.

The intra class coefficient (ICC) estimates and their 95% confident intervals (95% CI) for the acidic impacts at both survey times were calculated based on a single-rating, absolute-agreement, 2-way mixed-effects model. The following ICCs (95% CI) were found: acid impacts from drinks: ICC = 0.795 (0.605;0.900, *p* ≤ 0.001), acid impacts from food: ICC = 0.653 (0.381;0.822, *p* ≤ 0.001), all acid impacts: ICC = 0.809 (0.629;0.906, *p* ≤ 0.001), indicating a moderate to good reliability [12].

The kappa value for “I like to drink acidic drinks” at both survey times was 0.441 (*p* ≤ 0.01), and for “I like to eat acidic food” was 0.408 (*p* ≤ 0.01), indicating a moderate agreement [13] for both items (the combined Likert categories, see Section 2.4).

### 2.3. Tooth Wear Monitoring

Besides investigating the dietary behavior with a survey, tooth wear of one lower first molar (study tooth) was analyzed using an intraoral scanner (Trios 3, 3Shape, Copenhagen, Denmark). An experienced investigator (M.A.S.) scanned the study tooth at T0 and T1. To determine tooth wear, datasets of T0 and T1 were superimposed in an external 3D software (GOM Inspect, version V8 SR1, GOM GmbH, Braunschweig, Germany) using the established best-fit alignment with the iterative closest point technique for the measurement of the maximum vertical tissue loss in micrometer. The 3D measurements were conducted by a second investigator (M.B.S.) to avoid bias, and this investigator was blinded to dietary data.

### 2.4. Statistics

Of the 91 participants, 11 had some missing dietary data, and these were therefore not included. In total, the 80 complete datasets were analyzed here. For the quantitative data (tissue loss and the number of acid impacts), significant deviations from the Gaussian distribution were found (Kolmogorov–Smirnov test); therefore, these are given as the median and 95% confidence interval (after bootstrapping). Non-parametric test procedures were used for the analysis.

For taste preferences, the five-point Likert scale items were combined into three categories. For this purpose, the categories “I do not agree at all” and “I rather disagree” were pooled into “disagree”, and “I tend to agree” and “I fully agree” were pooled into “agree”. “Neither” remained a category of its own. In addition, the participants were divided into two groups according to the number of acid impacts per day (low acid impacts: <4 acid impacts per day; high acid impacts: ≥4 acid impacts per day) [14].

All statistical procedures were done with IBM SPSS Statistics version 27 (IBM Germany GmbH, Ehningen, Germany).

#### 2.4.1. Comparison of Dietary Data T0 to T1

The intra class coefficient (ICC) estimates and their 95% confidence intervals for the number of acidic impacts at T0 and T1 were calculated based on a single-rating, absolute agreement, 2-way mixed-effects model. The relation of taste preferences at T0 and T1 was analyzed using Kappa statistics.

#### 2.4.2. Comparison of the Number of Acid Impacts and Taste Preferences

The relation between the number of acid impacts and taste preferences at both T0 and T1 was analyzed using the Kruskal–Wallis test for independent samples.

#### 2.4.3. Comparison of Dietary Data with Tissue Loss Values

Qualitative data on dietary behavior and quantitative data on tissue loss values at both T0 and T1 were examined using the Kruskal–Wallis test for independent samples. Furthermore, Spearman’s rank correlation was computed to assess the relationship between acid impacts and tissue loss.

## 3. Results

### 3.1. Comparison of Dietary Data T0 to T1

Table 1 describes the acid impacts at the two observation time points (T0 and T1).

**Figure 1:**
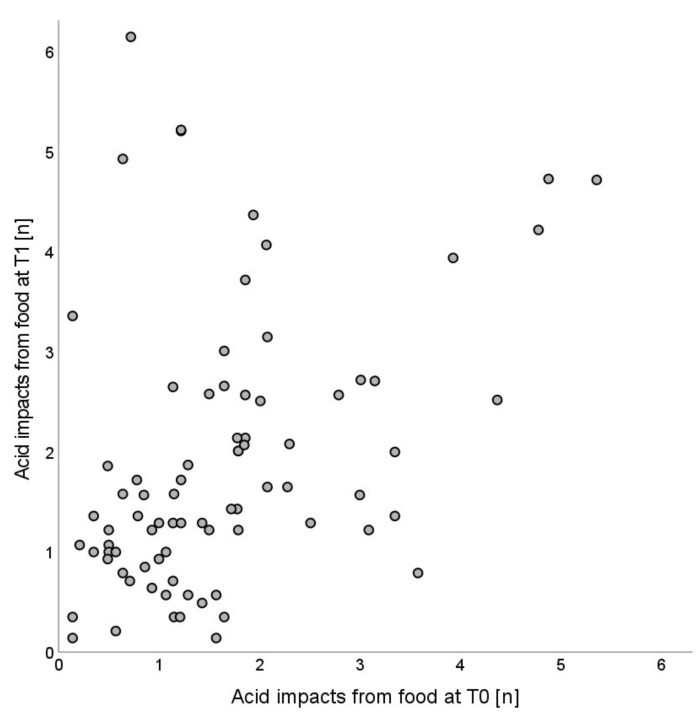
Scatterplot of acid impacts from food per day at baseline (T0) and at the end of the observation period after one year (T1).

The ICC for the comparison of the number of acid impacts from food, drinks, and overall at T0 and T1 was 0.387 (0.187;0.557, *p* ≤ 0.001), 0.068 (−0.154;0.284, *p* = 0.274), and 0.175 (−0.042;0.377, *p* = 0.057), respectively, indicating a fair relation for the first (Figure 1) and no significant relation for the others.

Kappa coefficients for “I like to eat acidic food” and “I like to drink acidic drinks” at T0 and T1 were 0.408 (*p* ≤ 0.01) and 0.403 (*p* ≤ 0.01), respectively, indicating a moderate relation. The comparison of the two items with each other resulted in a Kappa coefficient for T0 of 0.149 (*p* ≤ 0.05) and for T1 of 0.205 (*p* ≤ 0.05), indicating a slight relation.

**Figure 2:**
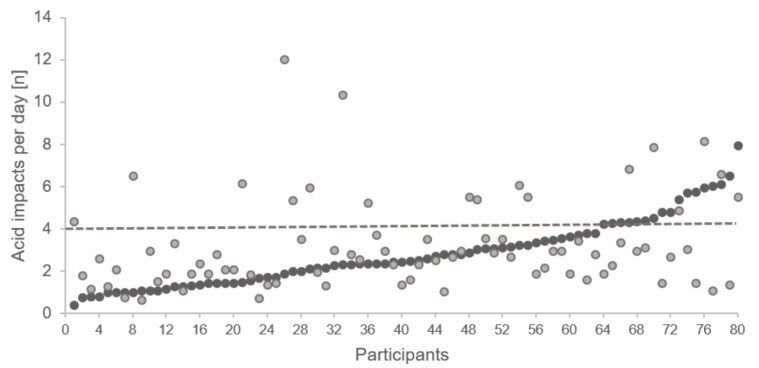
Scatterplot of acid impacts per day at T0 and T1, participants were ranked by their acid impacts at T0. The dotted line indicates the threshold for low and high acid impact groups; black dots indicate the number of acid impacts per day at T0 and the grey dots the corresponding values at T1.

At T0, 63 participants were in the low acid impacts group, and 17 were in the high acid impacts group; at T1, there were 62 and 18 subjects, respectively; 63.7% of the par-ticipants remained in the low acid impacts and 7.5% in the high acid impacts group, but 28.7% switched between groups. Figure 2 shows all participants ranked by the number of acid impacts per day at T0 and the corresponding data at T1.

### 3.2. Comparison of the Taste Preferences and Consumption Frequencies

Most items addressing taste preferences did not show a significant relation to the corresponding acidic impacts. Table 2 shows that those agreeing with the statement “I like to eat acidic food” did not have significantly more acid impacts from acidic food than those who were undecided or disagreed with this statement. This was true for both observation time points. In contrast, taste preferences regarding drinks had a significant effect on acid impacts, but this was only the case at T1.

### 3.3. Comparison of Dietary Data with Tissue Loss Values

The median tissue loss was 42.5 (38.0;49.0) µm with a range of 183 µm.

There was no significant correlation between acid impacts and tissue loss, regardless of whether the source of the acids (from drinks, food, or total) or the time point of the survey were considered. At T0, the correlation coefficients were 0.114 (*p* = 0.315), −0147 (*p* = 0.966), and −0.058 (*p* = 0.612), respectively, and at T1, they were 0.082 (*p* = 0.469), 0.005 (*p* = 0.966), and 0.018 (*p* = 0.871), respectively.

There was also no significant relationship between taste preferences for drinks and food and tissue loss. A numerical relationship was found between tissue loss and preference for acidic drinks at baseline. The tissue loss values were 39.0 (36.0;47.5), 44.5 (33.0;54.5), and 51 (38.0;67.9) µm for those who disagreed, were undecided, or agreed, but these differences did not reach statistical significance (*p* = 0.088).

## 4. Discussion

We investigated a new approach to shed light on the role of questionnaires in analyzing the relationship between diet and progression of tooth wear.

First of all, in the present context, there is no generally accepted questionnaire to fall back on. For this reason, we developed a new one and included some novel aspects. In this questionnaire, we collected the consumption frequencies of potentially erosive food and drinks that are frequently queried in the literature. In contrast to the usual analyses of individual drink or food categories with regard to their effect on tooth wear, we used the consumption frequencies only to determine the frequency of acid impacts overall. This approach seems more meaningful to us because the erosive potential of products in a category can vary greatly [15] (for example, different brands within the soft drink class), thus categories such as “soft drinks” or “fruit juices” do not represent an entity to which one could assign an independent erosive potential.

Dietary habits are multidimensional, of course, and self-reported frequencies may be biased in various ways, for example, in terms of recall, or social desirability [16]. We, therefore, included not only consumption frequencies but also qualitative aspects of diet and asked about taste preferences. We assumed that such questions could provide an indicator of basic preferences and general dietary behavior and might be more meaningful for our research question than consumption frequencies. In another context, food (taste) preferences have, indeed, been identified as an important factor in food choice [17].

Finally, we coupled our one-year wear observation period with two dietary surveys to obtain both a prospective and a retrospective dimension of dietary behavior in relation to the observation period.

With regard to the measurement of tissue loss, we focused on a single tooth observation, although tooth wear naturally can occur across the entire dentition. However, measuring tissue loss with intraoral scanners is less accurate when looking at larger sections of the jaw [18]. For this reason, we have only collected loss values for the first lower molar. This was well suited for our purpose because it is the tooth where tooth wear manifests itself earliest and has the highest progression rates [9,19,20].

First of all, we were interested in how consistent the data from the questionnaires were. With regard to the acid impacts from drinks and food, similar numbers were found at the beginning (T0) and at the end of the study (T1), but at the individual level, their correlation was low. That this was not due to random errors is indicated by the comparison with the results of the retest of our questionnaire, which showed a very good correlation with the consumption frequencies. This variability in the consumption frequencies is also reflected in the classification of the participants into the low and high acid impacts groups, as almost 30% of them were classified differently depending on whether the data from the beginning or the end of the studies were used.

Self-reported dietary data are subject to a variety of influencing factors, including memory, social desirability [7,16], and behavioral changes due to participation in a study. While the first two aspects mean that the actual dietary behavior differs from the reported one, the last aspect would imply an actual change in consumption. It is quite conceivable that our study participants, based on their knowledge of the study objective, have informed themselves about the reasons for tooth wear and the role of diet and have adapted their diet accordingly. This could have led to a reduction in reported acid impacts over time, but our data do not confirm this. That knowledge about a healthy diet only has a limited effect on behavioral change has also been shown in other contexts [21].

Eating habits are also a very strongly socially influenced construct [22], and it is quite conceivable that a dynamic social environment, as is possibly assumed especially in younger adults, could lead to variable and changing dietary behavior. Especially for the age group studied here, there seem to be many potential factors that determine eating behavior [23]. Among these factors are also taste preferences, and this factor is perhaps one for which a certain continuity can be assumed and which could have a certain basic influence on dietary behavior. It was, indeed, shown that those who agreed with the statement “I like the taste of most fruits” in fact ate more fruit than those who did not [24]. Furthermore, taste preferences and eating habits seem to be consistently associated with each other over a longer period of time [24]. In our study, taste preferences for both food and drinks at T0 and T1 were at least moderately consistent, in contrast to consumption frequencies. Contrary to what might be assumed, taste preference for drinks and food does not seem to be always in the same direction because those who liked to consume acidic drinks did not necessarily consume acidic food. Interestingly, taste preference was, except for acid drinks, only by trend associated with a corresponding frequency of consumption, which seems to contradict the results of the study mentioned above. However, it certainly depends on how the question is framed. In the context of erosive tooth wear, questionnaire items focus on drinks and food that are acidic in the chemical sense. However, low pH does not necessarily reflect taste sensation. For example, it was shown that participants rated the sweetness intensity of two commercial soft drinks with a sugar content of about 10% comparably high, even though the pH value of one drink was 6.3 and that of the other 2.1 [25]. It would, therefore, be interesting to investigate in further studies whether taste or consumption preferences can be found that have a better predictive value for tissue loss than the simple question about acidic taste sensations. Furthermore, the nutrient content and pH value may largely differ within a food category or even within a food item, and food items are often combined (e.g., fruits and yoghurt), which may influence the erosive potential. Finally, not only the original pH value of food is relevant for the erosive potential of food, but also the buffering capacity [26]. Moreover, human saliva has protective properties, thus a low pH value of food does not lead automatically to a low intraoral pH [26].

Our data show that, on the one hand, the participants reported a wide range in consumption of acidic food and drinks, and, on the other hand, they also experienced considerable tissue loss. This is similar to our first, very basic analysis, where we only looked at the dietary data reported at T0 [10]. However, neither the consumption frequencies of potentially erosive drinks or food nor the taste preferences at both time points could provide an explanation for the tissue loss. Accordingly, the classification of four or more acid impacts per day as a risk indicator for erosive tooth wear proposed by Lussi and Hellwig [14] could not be confirmed with our data; this is all the more so because about one-third of the participants changed this classification, depending on whether the data at T0 or T1 were taken into account. Thus, our study basically confirms the results of the two longitudinal studies mentioned at the beginning [6,7], which used a clinical index to assess tissue loss. Such indices represent a rather crude measure of tissue loss, and we had assumed that an objective and quantitative measure would be better suited to detect etiological factors attributable to diet. However, if we consider the heterogeneity of the dietary data at the two points in time of our study, it can be assumed that the dietary behavior, at least in the phase of life in which our participants found themselves, might be too variable to be grasped via only two data collection points. Thus, a cross-sectional survey of factors that can be attributed to diet might be generally unsuitable for explaining phenomena that manifest themselves over a longer period of time. A longer study period with multiple data collection points could shed light on this. Finally, however, the fundamental question can be asked whether and to what extent self-reported dietary habits have anything at all to do with real dietary behavior, and the relevance of dietary data collected on the basis of memory-based dietary assessment methods has generally been questioned [27]. As the relationship between diet and (oral) health remains an important area of research, it would be valuable to think about novel methods for assessing dietary behavior [28,29].

A strength of our study is that we have objectively quantified tissue loss with a digital measurement method that can represent tissue loss very sensitively. However, this relatively complex measurement method also means a limit to the number of test persons. The relatively small group of people is a limitation. Therefore, further studies must show how generalizable our results are. It could be regarded as a further limitation that other factors, such as oral hygiene behavior or bruxism, that could affect tooth wear were not included. Since it was not the aim of the present study to clarify which factors, in general, could be responsible for the wear that we found in our group of individuals, however, this limitation rather draws attention to methodological aspects for epidemiological studies in the field as a whole. If we consider bruxism, for example, as a possible factor for wear, studies show that self-reported behavior, collected with questionnaires, may also be less reliable in this context than one might presume [30]. It would, therefore, be an important research perspective to look at the research methodology for analytical, epidemiological questions on tooth wear from a broader perspective.

## 5. Conclusions

Considering the duration of longitudinal studies on tooth wear, it is important to note that the dietary behavior reported at intervals of only one year was only partially consistent. Although taste preferences seem to be more consistent over a longer period of time than consumption frequencies, it remains unclear to what extent these also reflect acid exposure from food and drinks. None of the dietary parameters showed a significant relation with tissue loss. The suitability of dietary questionnaires seems to be limited, and new survey methods may be needed to clarify the role of diet in the context of tooth wear.

## Figures and Tables

**Table 1:** Descriptive data of the number of acid impacts from different sources at the beginning (T0) and at the end (T1) of the observation period. Acid impacts from drinks included wine.

	Median	95% CI	Minimum; Maximum
Acid impacts from drinks T0	1.0	0.7;1.3	0.1;5.2
Acid impacts from drinks T1	0.9	0.7;1.1	0;11.7
Acid impacts from food T0	1.4	1.1;1.7	0.1;5.4
Acid impacts from food T1	1.5	1.3;1.9	0.1;6.2
Total acid impacts T0	2.5	2.3;3.0	0.4;7.9
Total acid impacts T1	2.7	2.3;3.0	0.6;12.0

**Table 2:** The number of acid impacts per day (median, 95% confidence interval, and range) in the different categories of taste preferences at baseline (T0) and follow-up after one year (T1) (* = Kruskal–Wallis test *p* ≤ 0.001). Drinks include wine.

	Disagree	Neither	Agree
	Acid impacts from food T0		
I like to eat acidic food T0	*n* = 27 1.2 (0.8;1.8) range 5.0	*n* = 15 1.2 (0.9;2.3) range 4.7	*n* = 38 1.5 (1.2;1.8) range 4.2
	Acid impacts from food T1		
I like to eat acidic food T1	*n* = 18 1.3 (0.9;2.1) range 4.6	*n* = 20 1.5 (0.8;2.1) range 5.0	*n* = 42 1.6 (1.3;2.2) range 5.8
	Acid impacts from drinks T0		
I like to drink acidic drinks T0	*n* = 50 0.9 (0.6;1.2) range 5.1	*n* = 14 1.1 (0.6;1.6) range 2.2	*n* = 16 1.5 (0.7;1.9) range 3.4
	Acid impacts from drinks T1		
I like to drink acidic drinks T1 *	*n* = 45 0.7 (0.5;0.9) range 5.1	*n* = 14 0.9 (0.6;1.1) range 2.7	*n* = 21 1.4 (1.2;1.7) range 11.2

## Data Availability

The datasets of this article are available from the corresponding author on a reasonable request.

## Appendix A

Questionnaire items

1. Frequencies

To investigate the number of acid impacts per day, the consumption of the following drinks and food was enquired in the questionnaire. The participants were asked to reflect on the last four weeks before answering the questions.

Non-alcoholic drinks:

- Energy drinks
- Lemonade
- Cola/cola mixed drinks
- Soft drinks other than cola and lemonade
- Light soft drinks
- Fruit juices
- Smoothies
- Iced tea
- Isotonic sports drinks
- Fruit tea

Food:

- Citrus fruits
- Soft fruits
- Stone fruits
- Pome fruits
- Pineapple
- Sour pickled food
- Salad dressings with vinegar
- Sour sweets

The following answer options were available, which were considered equivalent to the acid impacts per day in brackets:

- Never/rarely (0)
- 1–3x per month (0.07)
- 1–4x per week (0.36)
- 5–6x per week (0.79)
- 1–2x daily (1.5)
- 3–4x daily (3.5)
- ≥5x daily (5)

In addition, the consumption of wine was enquired with the following answer options available, which were considered equivalent to the acid impacts per day in brackets:

- Never (0)
- ≤1x per month (0.02)
- 2–3x per month (0.08)
- 1–3x per week (0.29)
- 4–6x per week (0.71)
- 1x daily (1)
- ≥2x daily (2)

The values of all items (or those related to food or drinks separately) were summed and constituted the total number of daily acid impacts (or acid impacts from drinks and food, respectively).

Example:

Consumption frequencies of a subject:

i. Energy drinks (1–4x per week)
ii. Smoothies (5–6x per week)
iii. Fruit tea (1–2x per day)
iv. Wine (4–6x per week)
v. Citrus fruits (1–2x daily)
vi. Soft fruits (1–2x daily)
vii. Salad dressings with vinegar (5–6x per week)
viii. Sour sweets (1–4x per week)

Calculation (addition of corresponding acid impacts):

i. 0.36
ii. 0.79
iii. 1.5
iv. 0.71
v. 1.5
vi. 1.5
vii. 0.79
viii. 0.36

Total: 7.51 acid impacts per day.

2 Taste preference

The answers to the questions were related to dietary behavior during the last four weeks.

1. I like to eat acidic food.
2. I like to drink acidic drinks.

The following answers could be chosen:

- I do not agree at all.
- I rather disagree.
- Neither.
- I tend to agree.
- I fully agree.
